# Watershed carbon yield derived from gauge observations and river network connectivity in the United States

**DOI:** 10.1038/s41597-023-02162-7

**Published:** 2023-05-13

**Authors:** Han Qiu, Xuesong Zhang, Anni Yang, Kimberly P. Wickland, Edward G. Stets, Min Chen

**Affiliations:** 1 Department of Forest and Wildlife Ecology, University of Wisconsin, Madison, WI 53706 USA; 2 USDA-ARS Hydrology and Remote Sensing Laboratory, Beltsville, MD 20705-2350 USA; 3 Department of Geography and Environmental sustainability, University of Oklahoma, Norman, 73019 USA; 4 Geosciences and Environmental Change Science Center, U.S. Geological Survey, Lakewood, CO 80303 USA; 5 U.S. Geological Survey, Mounds View, MN 55112 USA

**Keywords:** Carbon cycle, Hydrology

## Abstract

River networks play a critical role in the global carbon cycle. Although global/continental scale riverine carbon cycle studies demonstrate the significance of rivers and streams for linking land and coastal regions, the lack of spatially distributed riverine carbon load data represents a gap for quantifying riverine carbon net gain or net loss in different regions, understanding mechanisms and factors that influence the riverine carbon cycle, and testing simulations of aquatic carbon cycle models at fine scales. Here, we (1) derive the riverine load of particulate organic carbon (POC) and dissolved organic carbon (DOC) for over 1,000 hydrologic stations across the Conterminous United States (CONUS) and (2) use the river network connectivity information for over 80,000 catchment units within the National Hydrography Dataset Plus (NHDPlus) to estimate riverine POC and DOC net gain or net loss for watersheds controlled between upstream-downstream hydrologic stations. The new riverine carbon load and watershed net gain/loss represent a unique contribution to support future studies for better understanding and quantification of riverine carbon cycles.

## Background & Summary

Rivers and streams play a significant role in the global carbon cycle^1^. Recent studies estimated that 2.7–5.1 PgC yr^−1^ (including both organic and inorganic carbon in particulate and dissolved forms) is transferred from terrestrial ecosystems to river networks^2–5^; meanwhile rivers and streams emit ca. 1.8 PgC yr^−1^ into the atmosphere^6^ and export ca. 1.06 PgC yr^−1^ to estuaries including 0.238 PgC yr^−1^ of dissolved organic carbon (DOC) and 0.244 PgC yr^−1^ of particulate organic carbon (POC)^7^. In addition, lakes/reservoirs that are interspersed along river networks are also important modifiers of the global carbon cycle. For example, lakes/reservoirs can fix 0.376 PgC yr^−1^ ^8^, bury ca. 0.15 PgC yr^−1^ ^9^ and release 0.75–1.65 PgC yr^−1^ ^10^. The magnitude of carbon stocks and fluxes in river networks are comparable to other major components of the global carbon cycle, such as the terrestrial C sink of ca. −3.4 ± 0.6 PgC yr^−1^ (negative sign means C fluxes from the atmosphere to land) or the oceanic sink of 2.5 ± 0.9 Pg C yr^−1^ ^11^. However, the current estimates of carbon budgets of river networks are subject to large uncertainties^12^, limiting effective management of carbon to mitigate negative climate change impacts. Therefore, there is an urgent need for new datasets to support better understanding and quantification of carbon stocks and fluxes related to river networks.

The estimates of carbon emissions into the atmosphere and carbon burial along river networks are often derived by extrapolating site-scale observations to regional scales, and therefore are subject to large uncertainties^12^. In contrast, the estimation of global and continental riverine carbon export to coastal waters is of high confidence thanks to extensive observations of carbon concentration and streamflow data near river mouths^1,3^. The early estimate of the global riverine carbon export of 0.9 PgC yr^−1^ ^13^ that was derived nearly four decades ago has been widely used in studies constraining the global carbon cycle^2,5,10,14^. Recent updates only slightly increased the global riverine carbon export to 0.95 PgC yr^−1^ ^4^ and 1.06 PgC yr^−1^ ^7^. In the Conterminous United States (CONUS), previous studies also estimated the export of carbon at the outlets of large watersheds^15^ and showed large amounts of riverine carbon exported to the coastal region. However, different reaches receive different amounts of carbon loads (i.e., terrestrial-derived carbon and upstream loads) and function differently in removing carbon from the water column (i.e., burial and outgassing). The net balance of those inputs and outputs determines whether a river reach gains (downstream export – upstream load >0) or loses (downstream export – upstream load <0) carbon. Although the global/continental scale riverine carbon cycle studies demonstrated the significance of rivers and streams for linking land and coastal regions, the lack of spatially distributed riverine carbon load data represents a gap for quantifying riverine carbon net gain or net loss in different regions, understanding mechanisms and factors that influence riverine carbon cycling, and testing simulations of aquatic carbon cycle models at a refined scale.

**Fig. 1:**
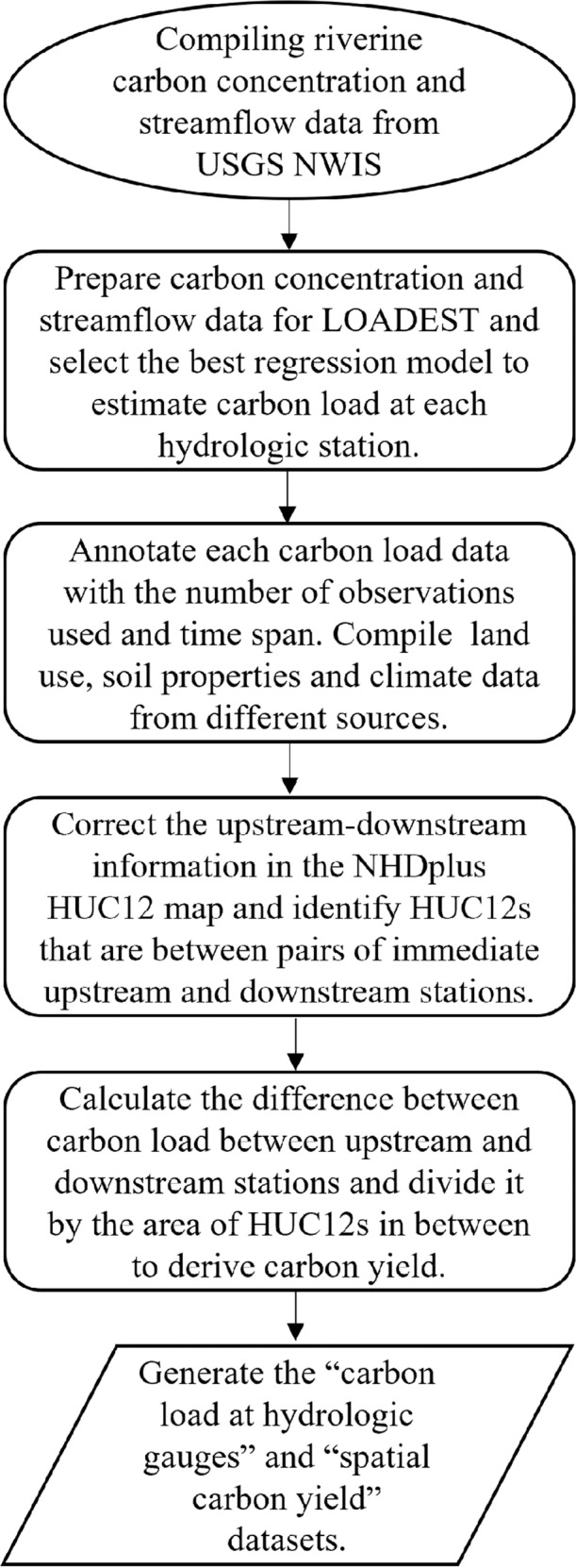
Schematic overview of the workflow for generating carbon load and yield data across the CONUS.

The workflow of this data describer is shown in Fig. 1. Here, we derived riverine loads of particulate organic carbon (POC) and dissolved organic carbon (DOC) for over 1,000 hydrologic stations across the CONUS and further use the upstream-downstream drainage information from the National Hydrography Dataset Plus (NHDPlus; https://nhdplus.com/NHDPlus/NHDPlusV2_data.php) to estimate the net gain or net loss of POC and DOC between hydrologic stations. The newly derived riverine carbon load dataset and spatially distributed information regarding riverine carbon net loss and net gain are expected to inform future studies for understanding controls of riverine carbon cycle and an independent dataset for model verification. Key methods and procedures used to develop the datasets are described in the “Methods” section.

## Methods

### Compiling riverine organic carbon observations and deriving carbon load

Daily stream flow and POC and DOC concentration data were obtained from the United States Geological Survey (USGS) National Water Information System (NWIS; https://waterdata.usgs.gov/nwis/dv/?referred_module=sw) through November 2014. We paired the carbon concentration and streamflow data that occurred on the same day and calculated riverine POC and DOC load using the Load Estimator Model (LOADEST)^16^. LOADEST is a FORTRAN program that uses Adjusted Maximum Likelihood Estimation (AMLE)^17^ to determine coefficients of a regression model and estimate the load of a constituent based on the time series of streamflow and constituent concentrations. We retained only stations that possessed at least 12 observations for further analysis. Note that, the use of 12 observations meets the requirement by LOADEST to derive valid regression functions, but the limited number of observations may not accurately estimate the riverine carbon fluxes that are influenced by numerous terrestrial and aquatic carbon cycling processes. Therefore, users may use a larger number of observations to select gauges to support their studies. In total, we compiled 62,488 DOC concentration data from 1249 stations, and 36289 POC concentrations from 900 stations. The time frame and number of observations are provided in the shared data products. It is worth noting that riverine carbon not only contains POC and DOC, but also particulate inorganic carbon (PIC) and dissolved inorganic carbon (DIC), which combined can account for half or even more of the total riverine carbon^15,18^. As the direct measurements of PIC and DIC are relatively scarce compared to organic carbon observations, here we focus on organic carbon. In addition, the POC data analysed in this study are bio-spheric and do not include petrogenic sources, which could further increase the amount of POC by more than 20%^19^.

**Fig. 2:**
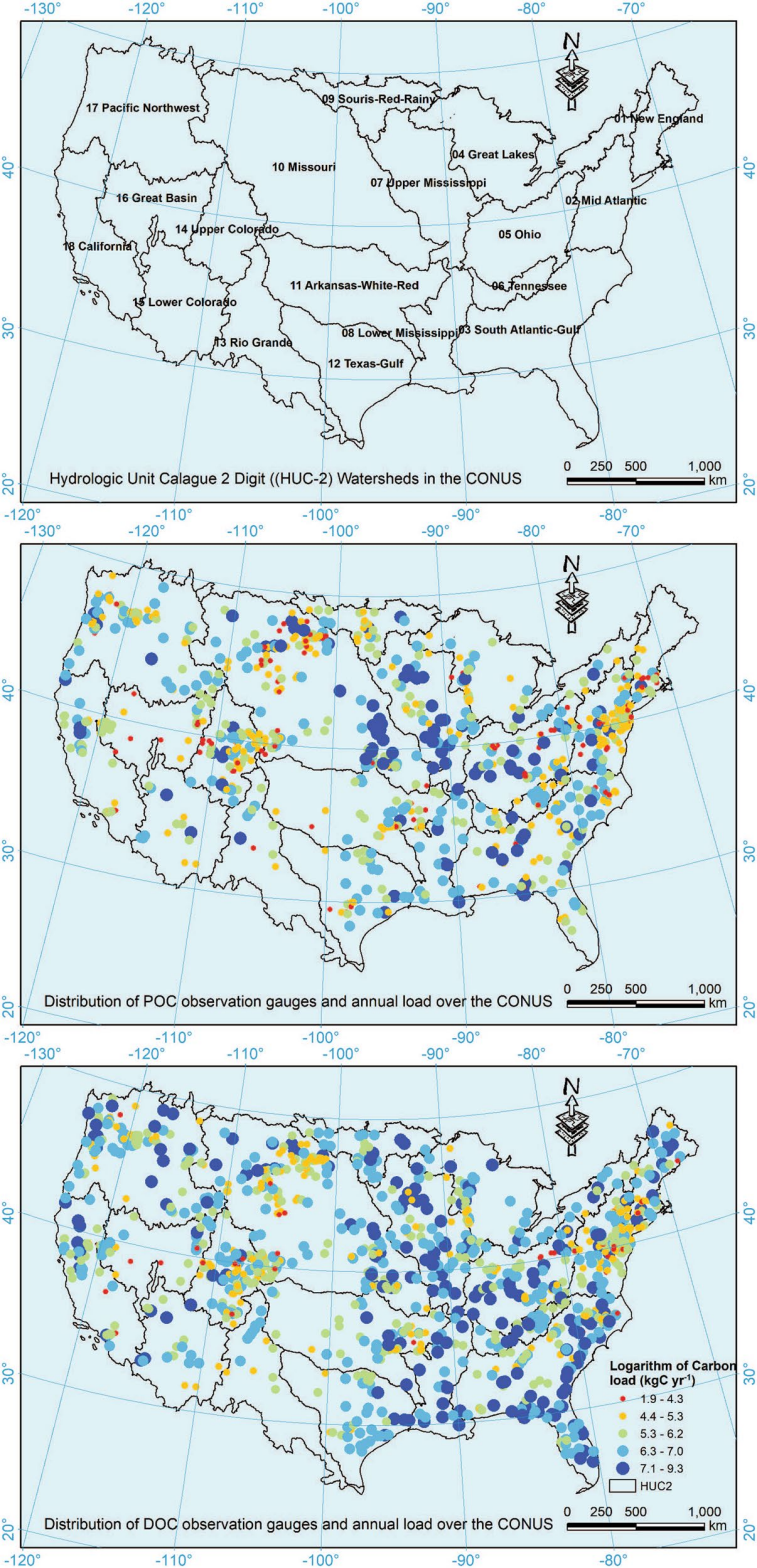
Spatial distribution of hydrologic stations used to calculate riverine load of POC and DOC over the CONUS. The boundary line shows the Hydrologic Unit Catalogue 2-digit (HUC2) watersheds (https://prd-tnm.s3.amazonaws.com/index.html?prefix=StagedProducts/Hydrography/WBD/HU2/Shape).

For each station and constituent, we fitted 9 candidate regression models within LOADEST (Table 1) and chose the one with the least Akaike Information Criteria (AIC)^16^ value to estimate riverine carbon load. AIC considers not only the likelihood of a model measured by the difference between observations and model prediction, but also the number of parameters used in the model^20^. AIC prefers parsimonious models and has been widely used in model selection to avoid overfitting^21^. Figure 2 shows the geographic distribution of the stations used to derive riverine POC and DOC load, as well as the estimated annual mean load for each station.

**Table 1:** Regression models used in LOADEST.

Model ID	Regression models
1	*a*_0_ + *a*_1_·In *Q*
2	*a*_0_ + *a*_1_·In *Q* + *a*_2_·In *Q*^2^
3	*a*_0_ + *a*_1_·In *Q* + *a*_2_·*dtime*
4	*a*_0_ + *a*_1_·In *Q* + *a*_2_·sin(2*π*·*dtime*) + *a*_3_·*cos*(2*π*·*dtime*)
5	*a*_0_ + *a*_1_·In *Q* + *a*_2_·In *Q*^2^ + *a*_3_·*dtime*
6	*a*_0_ + *a*_1_·In *Q* + *a*_2_·In *Q*^2^ + *a*_3_·sin(2*π*·*dtime*) + *a*_4_·*cos*(2*π*·*dtime*)
7	*a*_0_ + *a*_1_·In *Q* + *a*_2_·sin(2*π*·*dtime*) + *a*_3_·*cos*(2*π*·*dtime*) + *a*_4_·*dtime*
8	*a*_0_ + *a*_1_·In *Q* + *a*_2_·In *Q*^2^ + *a*_3_·sin(2*π*·*dtime*) + *a*_4_·*cos*(2*π*·*dtime*) + *a*_5_·*dtime*
9	*a*_0_ + *a*_1_·In *Q* + *a*_2_·In *Q*^2^ + *a*_3_·sin(2*π*·*dtime*) + *a*_4_·*cos*(2*π*·*dtime*) + *a*_5_·*dtime* + *a*_6_·*dtime*^2^

Where ln *Q = *ln(*streamflow*) - center ln(*streamflow*); *dtime = *decimal time - center of decimal time; *a*_0_, *a*_1_, *a*_2_, *a*_3_, *a*_4_, *a*_5_ are regression coefficients.

### Calculating drainage area of each hydrologic station from NHDplus

**Fig. 3:**
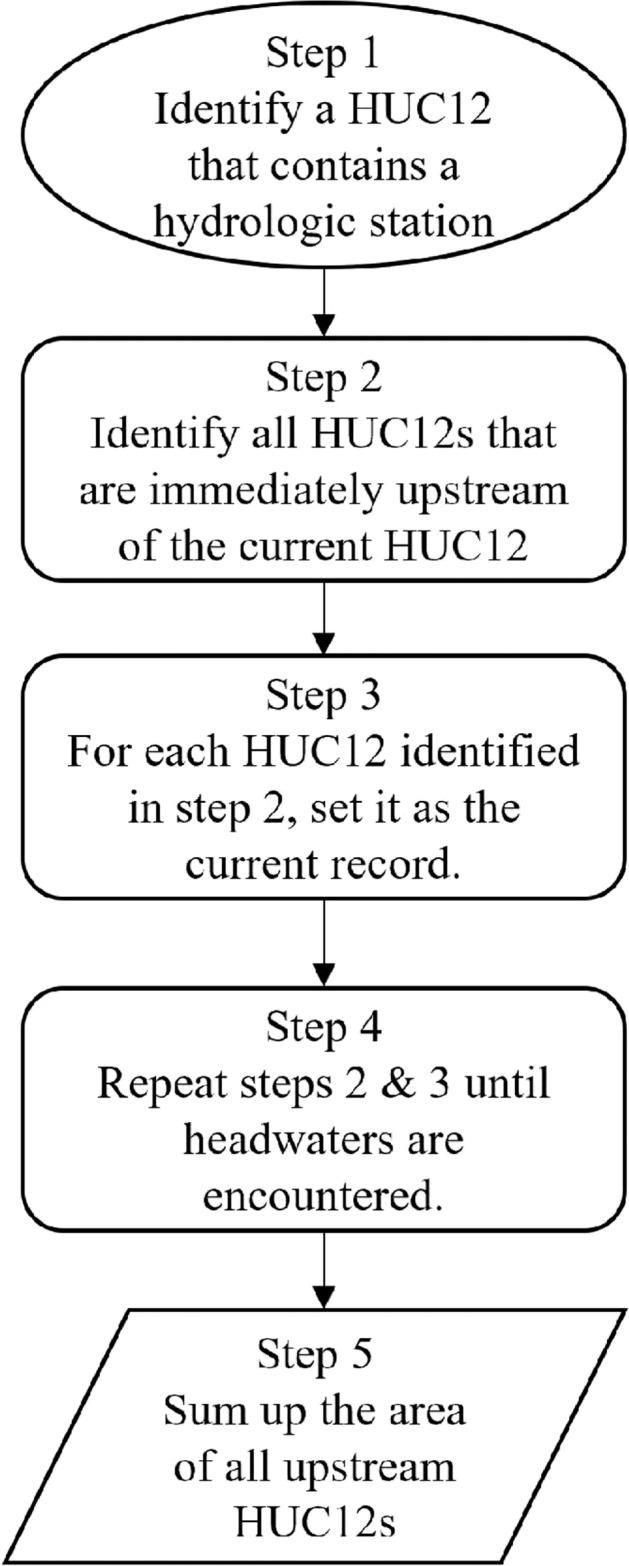
Flow chart of deriving the hierarchical upstream-downstream structure of HUC12 polygons and the drainage area for each hydrologic station.

Drainage area is an important property that influences the behavior of a watershed and load of riverine carbon^22^. For most hydrologic stations used here, the drainage area information is available from USGS Geospatial Attributes of Gages for Evaluating Streamflow (GAGES-II) dataset^23^. For the remaining stations, we estimated their drainage area using the upstream-downstream topology information contained in the NHD-Plus hydrologic unit dataset. A hydrologic unit is a small catchment area for a segment of the river networks. At the hydrologic unit catalogue 12-digit (HUC12) level, there are a total of 86,744 hydrologic units over the CONUS with an average size of ca. 104 km^2^. For each HUC12, we used “ToHUC” to identify all HUC12 drains into it, and further traced upstream to all HUC12s that drain to a comment outlet (Fig. 3). The areas of all the HUC12 that drain to a HUC12 are summed up as the drainage area of that HUC12. While calculating drainage area for the hydrologic stations, we found that the “ToHUC” field does not always match the actual downstream HUC12 as visually identified using the NHDplus river network flowlines. This error can cause large bias in estimated drainage area for multiple hydrologic stations. Therefore, we used the riverine flowline network to manually correct the “ToHUC” field to ensure complete accounting of all HUC12s that drain to a hydrologic station. The updated “ToHUC” information for each HUC12 catchment area is included within the newly developed “watershed carbon yield” dataset in this study.

With the procedures outlined in Fig. 3, we calculated the drainage area of the stations used to analyse riverine load and yield of DOC and POC. Note that for those stations that falls within headwater HUC12s, we used USGS reported drainage area instead of the area of HUC12 they are located within, as those stations only control a fraction of a headwater HUC12 and using the entire area of the HUC12 could substantially overestimate the drainage area.

### Deriving spatially distributed carbon yield

**Fig. 4:**
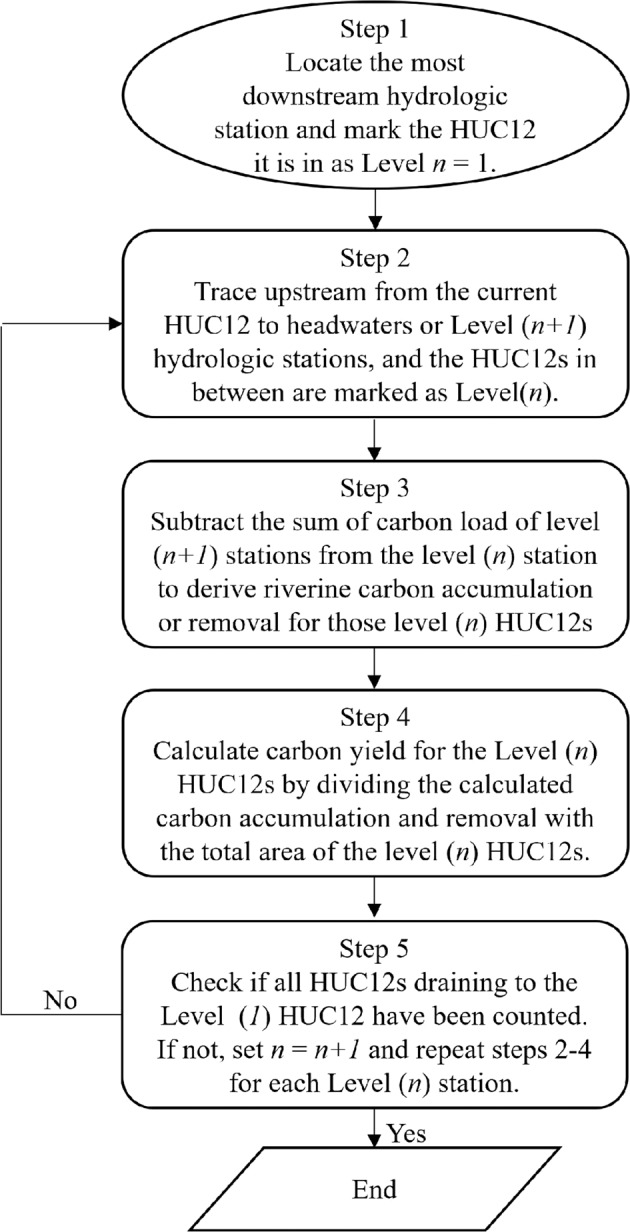
Procedures used to identify HUC12s between upstream and downstream hydrologic stations and calculate carbon yields for those HUC12s. For each HUC2 watershed (Fig. S1), we identify one or multiple stations that do not drain to any downstream stations and mark them and HUC12 they are located within as Level (*1*). Higher levels of HUC12s are further identified based on the outlined procedures. Depending on the number of HUC12s contained in a watershed and the number of stations with observations, the number of levels of HUC12 vary substantially. The HUC12s with the same level and located within the same HUC2 share the same value of carbon yield.

**Fig. 5:**
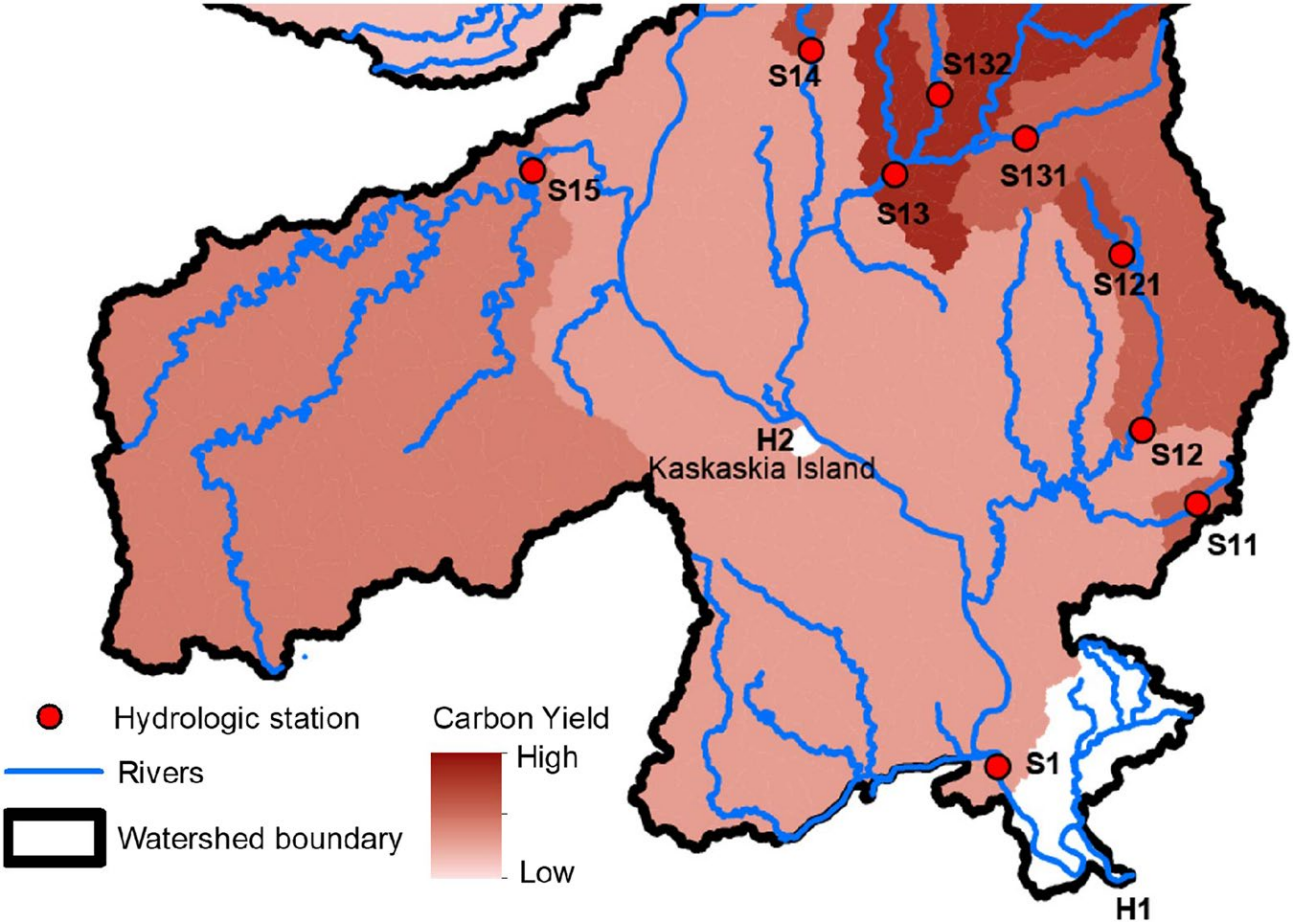
Illustration of the calculation of spatially explicit carbon yield by combining station observed carbon load and the upstream-downstream topology of HUC12s. H1 is the outlet of the watershed. H2 is a “Closed Basin” HUC12 that does not drain to any other HUC12s. The HUC12s between S1 and H1 are marked as “no data” (or white) because there are no stations downstream of S1 that allow us to calculate the changes in carbon load between S1 and H2. The HUC12 where H2 is located is also marked as “no data” because they are “Closed Basin” and do not drain to the common outlet S1.

Using the carbon load data at the hydrologic stations and the upstream-downstream topology information that links HUC12s, we applied the procedures outlined in Fig. 4 to calculate carbon yield of DOC and POC for each HUC12. In doing so, we used the upstream-downstream routing sequence data from the Watershed Boundary Dataset (WBD; https://www.usgs.gov/national-hydrography/access-national-hydrography-products) We corrected the topology information contained in the “ToHUC” field of the WBD dataset to ensure they are aligned with the upstream-downstream routings sequence from the NHDplus flowlines. We started with locating the most downstream HUC12 polygon that contains a hydrologic station, marking it as L(*1*) (or the outlet HUC12). From the L(*1*) HUC12, we traced upstream until encountering hydrological stations and marked those stations as L(*2*). All HUC12s that are upstream of the L(*1*) station and downstream the L(*2*) stations are marked with L(*2*). From the L(*2*) stations, we further traced upstream to L(*3*) stations and marked L(3) HUC12s. The above procedures are repeated until all headwaters are traced. We further calculated the L(*n*) carbon load as the sum of all L(*n*) hydrologic stations and the L(*n*) drainage area is the sum of the L(*n*) HUC12s. Eventually, we calculate the carbon yield of the L(*n*) HUC12s using the following equation.

1 $${Y}_{n}=\frac{\sum {F}_{n-1}-\sum {F}_{n}}{\sum {A}_{n-1}-\sum {A}_{n}}$$

where *Y*_*n*_ is the carbon yield of L(*n*) HUC12s; *F*_*n*_ and *F*_*n-*1_ are the carbon load of a L(*n*) station and its upstream L(*n*-1) stations, respectively; and *A*_*n*_ and *A*_*n-*1_ are drainage areas of a L(*n*) station and the sum of the drainage areas of its upstream L(*n* -1) stations. In Fig. 5 we visually illustrate how we calculated the spatially distributed carbon yield. In Fig. 5, S1 is the most downstream or L(*1*) hydrologic station. S11, S12, S13, S14, and S15 are the L(*2*) stations that are immediately upstream of S1. S121 is a L(3) station that is upstream of S12, while S131 and S132 are L(*3*) stations upstream of S13. All HUC12s that are upstream of the L(1) station and downstream of its L(*2*) stations are L(*1*) HUC12s and share the same carbon yield as denoted by the same colour. The carbon yield for the L(*1*) HUC12s is calculated as [F_1_ – (F_11_ + F_12_ + F_13_ + F_14_ + F_15_)]/ [A_1_ – (A_11_ + A_12_ + A_13_ + A_14_ + A_15_)]. Likewise, the HUC12s between a L(*2*) station and its immediate upstream L(3) stations are marked as one group of HUC12s that have the same carbon yield. For example, the carbon yield for HUC12s upstream of S12 and downstream of S121 is calculated as (F_12_ – F_121_)/(A_12_-A_121_). The carbon yield for all the HUC12s upstream of S121 is directly calculated as F_121_/A_121_ since there are no sampling stations upstream of S121. The same procedures are used to calculate carbon yield of every HUC12 that drains to the L1 station.

Note that, in the process of calculating watershed carbon yield, we excluded those HUC12s that drain to a “Closed Basin” HUC12. For example, within the region shown in Fig. 5, there is a “closed basin” HUC12 (Kaskaskia Island), and we excluded all HUC12s draining to it from our analysis by assuming water and carbon cycles of those closed watersheds do not actively interact with other basins.

### Spatial distribution of carbon yield

**Fig. 6:**
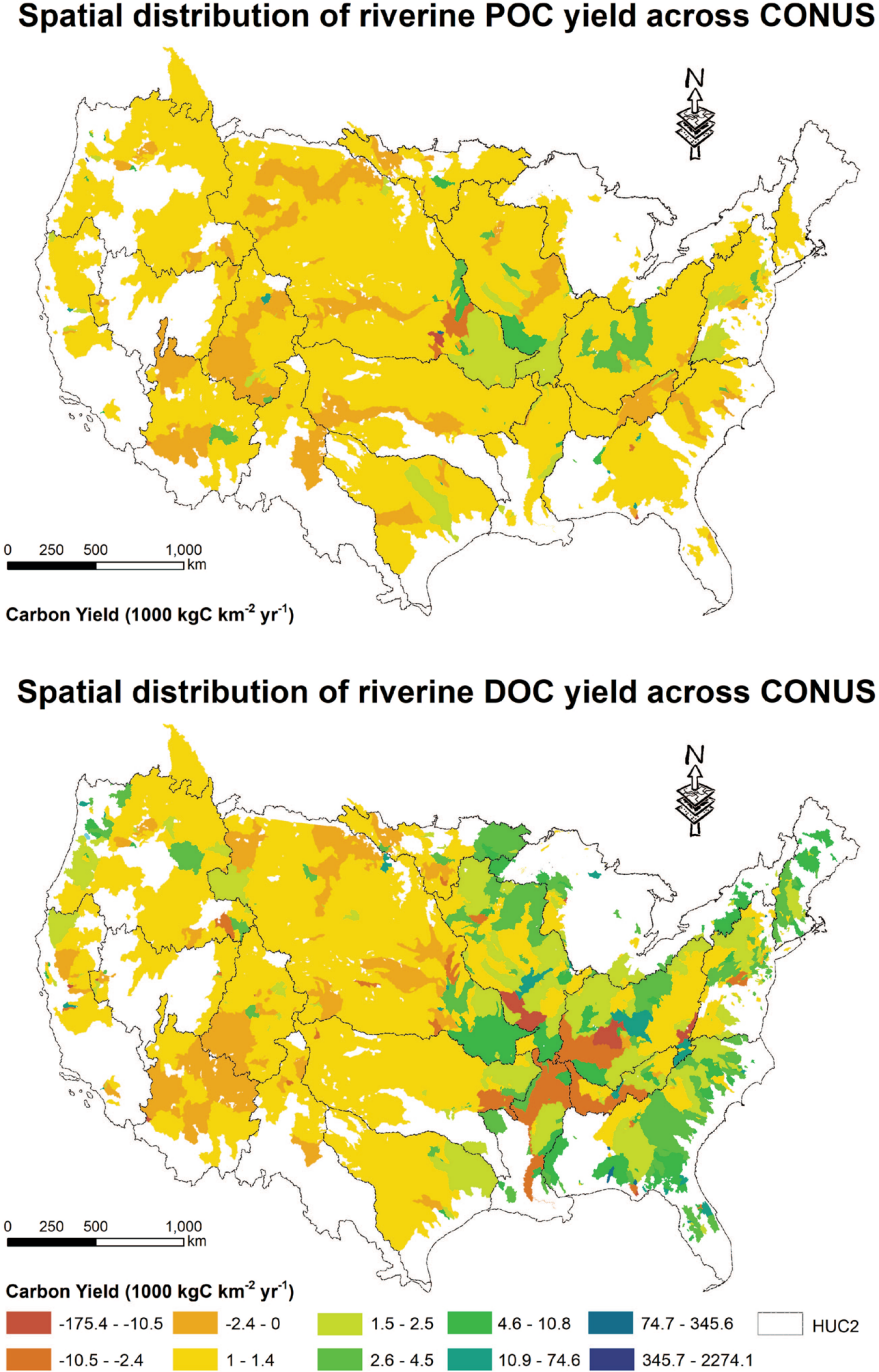
The spatial distribution of carbon yield for particulate organic carbon (**a**) and dissolved organic carbon (**b**) over the conterminous United States.

The estimated yield of POC and DOC for the HUC12s controlled by hydrologic stations with estimated carbon load (Fig. 2) is visualized with the ArcGIS software (10.7) as shown in Fig. 6. It is worth noting that the empty (or no-data) areas in Fig. 2 are mainly caused by the lack of concurrent carbon and streamflow data at hydrologic stations that control those empty areas. It is also possible that some hydrologic stations are in tidal areas, making it difficult to map their drainage area. In addition, transboundary water transfer could be another factor, which deserves future analysis.

By dividing the carbon load at a station with its drainage area, the spatially averaged carbon yield for the station’s upstream area is obtained. That value is always greater than 0. Such a method ignores the variability in the role of different regions for processing POC and DOC. With the newly generated “watershed carbon yield” map, we can further examine the HUC12s contributing to the net gain of carbon load, and those removing carbon.

## Data Records

The data products are shared at figshare.com^24^, including three datasets: (1) the “POC load at hydrological stations” and “DOC load at hydrological stations” and (2) the “watershed carbon yield” dataset. The “POC/DOC load at hydrological stations” dataset contains the carbon load data at each hydrological station (Table 2). For DOC, the stations with load greater than 1.4 × 10^8^ kgC yr^−1^ are mostly located in the Mississippi River Basin while the Pacific Northwest, Souris-Red-Rainy, and South Atlantic-Gulf regions each contain one station with load greater than 1.4 × 10^8^ kgC yr^−1^ (Fig. 2). For POC, most stations have a load less than 1.4 × 10^8^ kgC yr^−1^, except for five stations within the Mississippi River Basin. The ‘USGS-07295100’ station is near the outlet of the Mississippi River Basin, with a drainage area of ca. 2.9 million km^2^. This station has the largest load of POC and DOC (1.0 × 10^9^ kgC yr^−1^ and 1.8 × 10^9^ kgC yr^−1^, respectively). The number and timespan of available observations, the regression model selected to estimate carbon load, and the R-square value (calculated using all the historical data used to fit the LOADEST model and LOADEST predictions) measuring the performance of the selected regression model are also provided. Users can select hydrologic stations with R-square values that meet their own standards to support different analyses. The data used to derive our results are distributed over 1970s (12%), 1980s (15%), 1990s (35%), 2000s (27%), and 2010s (11%).

**Table 2:** Data records contained in the “POC load at hydrological stations” and “DOC load at hydrological stations” datasets.

Field name	Definition
Station ID	U.S. Geological Survey designated ID
NHDplus derived drainage area	Drainage area controlled by the hydrologic station (km^2^)
USGS reported drainage area (km^2)	Drainage area controlled by the hydrologic satiation (km^2^). Derived from USGS GAGEII Dataset.
Drainage area data source flag	0 means no USGS record
Carbon load	The amount of carbon load from the hydrologic station (gC day^−1^)
Number of observations	The number of paired carbon concentration streamflow data
Data period	The starting and ending years of the observed data
Regression model	The ID of one of the nine LOADEST regression models
R-square(%)	Percent of variance explained by the selected LOADEST regression model

The “watershed carbon yield” dataset contains the yield of POC and DOC for each HUC12 (Table 3). This dataset can be linked with other information from NHDplus and WBD dataset at the HUC12 level to further expand the existing national hydrography databases. In general, the stations included in this study range from 0.18 km^2^ to nearly 3 million km^2^. Small watersheds (<100 km^2^) are distributed across every Hydrologic Catalogue Unit 2-digit (HUC2) watershed in the CONUS (Fig. 2), while large drainage area (>10,000 km^2^), particularly those >100,000 km^2^, are mainly distributed in the western and middle US, such as the Columbia, Colorado, and Mississippi river basins. In the eastern US, there are numerous small watersheds. For DOC, there are 53,991 (totalling 5,021,436 km^2^) HUC12s with carbon yield greater than 0, while 9,009 (totalling 851,277 km^2^) HUC12s with negative carbon yield. For POC, 52,883 HUC12s (totalling 4,915,709 km^2^) net gain POC while 7,833 HUC12s (totalling 750,595 km^2^) remove POC.

**Table 3:** Data records contained in the “watershed carbon yield” dataset.

Field name	Definition
HUC12 ID	12-digit ID for each HUC from the NHDplus dataset
ToHUC	The HUC12 ID that current HUC12 drains to
Area	The area of current HUC12 (km^2^)
POC yield	Yield of particulate organic carbon (kgC km^−2^ year^−1^)
DOC yield	Yield of dissolved organic carbon (kgC km^−2^ year^−1^)

In general, ca. 85% and 86% of the HUC12s remove DOC and POC, respectively. Therefore, it is reasonable to assume that the carbon yield from small watersheds is less than the amount of carbon transported from land to rivers. Previous studies estimated POC load based on soil erosion models and topsoil SOC content and suggested substantial uncertainties with those estimates^25–27^. To date, there is still a lack of direct estimates of DOC transport from land to rivers. The spatial maps of POC and DOC could serve as a lower bound estimate of carbon transported from land to rivers, particularly for those small watersheds that are subject to minimal riverine processes.

## Technical Validation

**Fig. 7:**
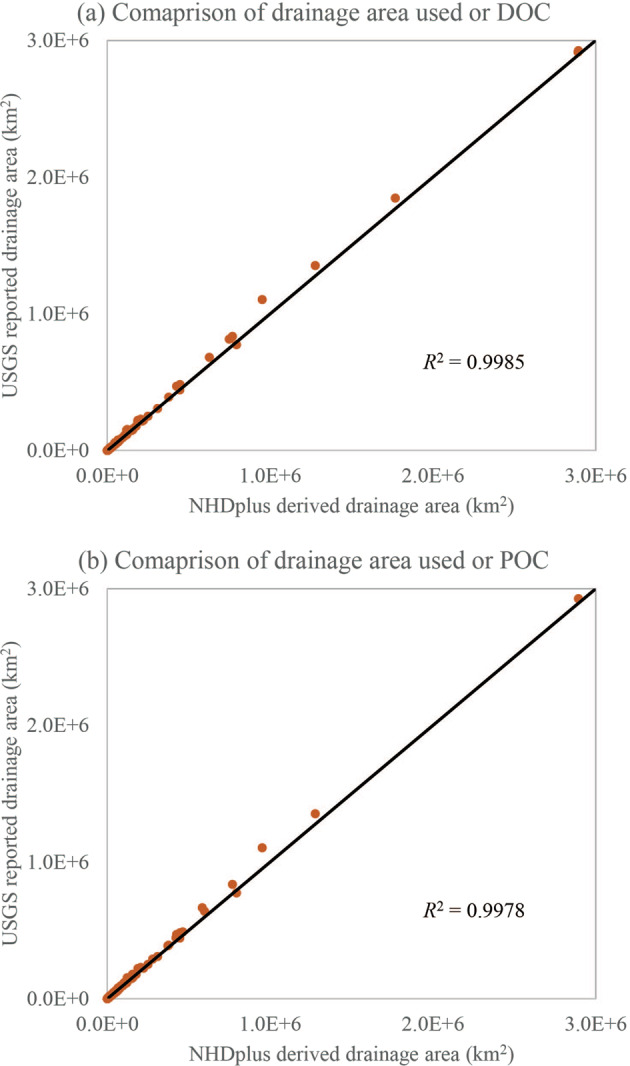
Comparison between drainage area derived from NHDplus and reported by USGS for (**a**) DOC and (**b**) POC. Line indicates 1:1 relationship.

We used the upstream-downstream topology information contained in NHDplus to derive drainage area for stations without USGS reported drainage area. To assess the validity of those estimates, we compared the NHDplus derived drainage area for those stations that have USGS reported drainage area (Fig. 7). For DOC, 1209 out of 1250 stations have both USGS reported and NHDplus derived drainage area. For POC, 887 out of 898. The high R-square value indicates the NHDplus derived drainage area highly agrees with the USGS reported values. This result also confirms that the upstream-downstream topology information that we have corrected for NHDplus HUC12s is reliable.

**Fig. 8:**
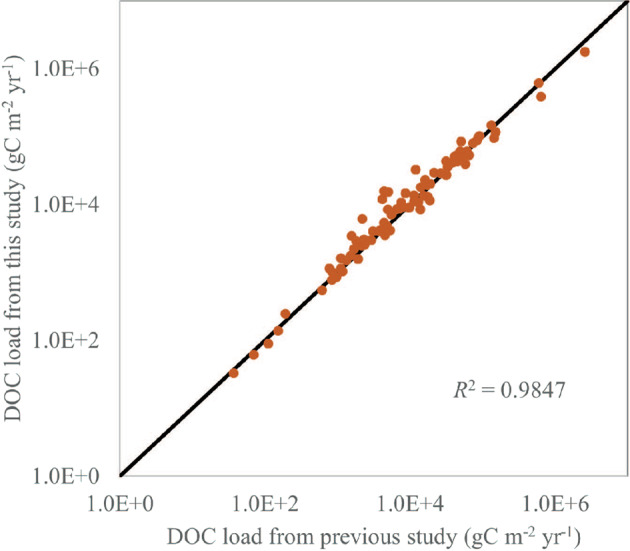
Comparison between DOC loads estimated in this study and those from a previous study^15^.

Stets and Striegl^14^ used LOADEST to estimate carbon exports by large watersheds within the CONUS to the coastal region. In their study, a total of 95 sites were used for DOC. The DOC concentration and streamflow data used by Stets and Striegl^14^ were also obtained from the USGS NWIS database. Therefore, their estimates can be used as a validation dataset to assess the quality of our calculation. We extracted the calculated DOC loads from our datasets for those sites reported in Stets and Striegl^14^. The comparison results show a high agreement between the two datasets (Fig. 8). The small deviations are likely due to the use of carbon concentration and streamflow data from different time periods. Our study used all data up to 2014, which extend beyond the period used in Stets and Striegl^14^. Overall, the comparison corroborates the validity of our estimates of carbon load at the hydrologic stations.

Collectively, the above technical validation of the drainage area derived from NHDplus HUC12s and carbon load estimated from carbon concentration and streamflow data justify the robustness of the data processing and analysis procedures. As such, the quality of the watershed carbon yield maps that were derived based on the above two datasets are assured.

It is worthing noting that using a small number of observations to estimate carbon loads could be subject to uncertainties. Here, we conducted further analysis to identify 48 pairs of upstream and downstream hydrologic stations for POC and 92 pairs for DOC. In doing so, we set a criterion of 20 or fewer observations for the upstream stations and 30 or more observations for the downstream stations. Then we inversed the upstream POC/DOC loads by multiplying the downstream POC/DOC loads by the ratio between the drainage area of the upstream station and that of the downstream station. The assumption here is that the POC/DOC loads estimated with more observations are more reliable than those estimated with fewer observations. Comparing the inversed and LOADEST-estimated POC/DOC loads at hydrologic stations with fewer than 20 observations helps verify if the estimates derived with a small number of observations are robust.

**Fig. 9:**
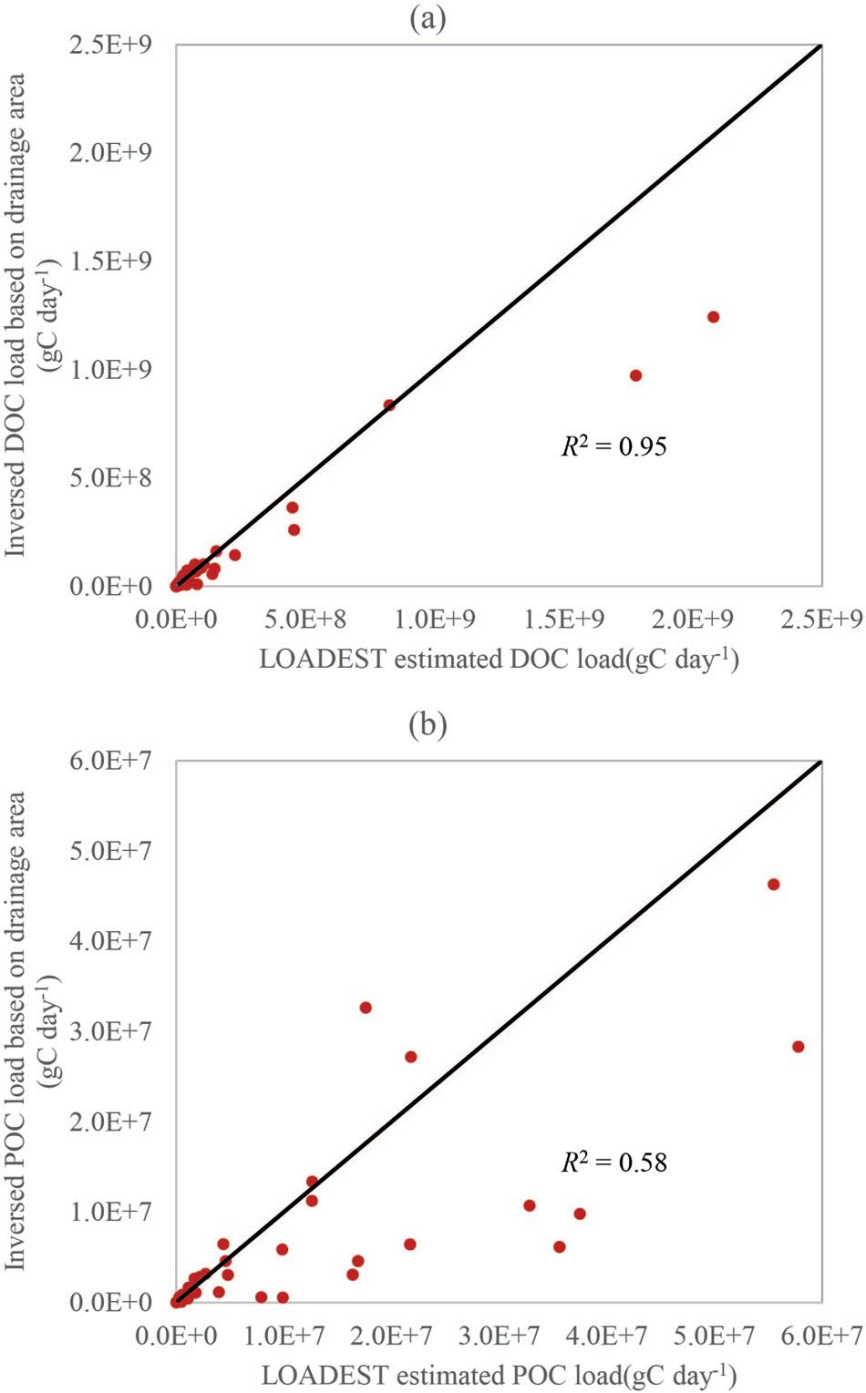
Scatter plots of inversed and LOADEST estimated DOC (**a**) and DOC (**b**) loads.

We observed a positive correlation (0.95 for DOC and 0.58 for POC) between the inversed and LOADEST-estimated DOC/POC loads (Fig. 9), indicating that drainage area is a major factor controlling DOC/POC loads. Note that the slope of the regression lines is less than 1 for both POC and DOC. This suggests that, in addition to drainage area, other factors (such as land use, climate, and hydrologic conditions) could further confound the relationship between POC/DOC loads at upstream and downstream stations. Overall, the high correlation for DOC justifies using fewer than 20 observations, which would not cause much loss of accuracy. For POC, using a small number of observations could cause certain loss of accuracy, as indicated by the spread of the scatter points. As POC loads could also be influenced by other factors besides drainage area, future research is necessary to identify and consider other factors to further confirm the credibility of POC loads estimated with a small number of observations. Given current evidence, we recommend using caution when estimating POC/DOC loads with LOADEST using a small number of observations, particularly for POC.

## Usage Notes

We encourage interested users to read the methods and data records sections to understand how the data are derived and organized. Users can select a subset of CONUS scale dataset to meet their research and application purposes at smaller spatial scales. Also, as the carbon loads and yields were derived for different time periods based on availability of observed carbon concentration and streamflow data, we suggest users select hydrologic stations that contain data representative to the time periods of their studies. In general, the new datasets generated from this study could be used to support, but not limited to, the following types of efforts:

1. The “carbon load at hydrologic stations” dataset can be used to support model development to simulate riverine carbon load across the CONUS. The estimated carbon load data at the hydrological stations is useful for validating watershed models^28,29^ that can represent the coupled terrestrial and aquatic carbon cycle.
2. The carbon load data is distributed across a wide range of watersheds (Fig. 1) with varied climate, land use, terrain, and soil properties. Also, the hydraulic properties (e.g., length, width, depth, and flow rate) of reaches can vary greatly from upstream to downstream. In addition to the terrestrial and aquatic properties form the NHDplus database, users can further collect or compile additional watershed properties from other data sources (e.g. “Mainstem Rivers of the Conterminous United States”^30^) to complement the current data records and analyse environmental controls of riverine carbon load.
3. The “watershed carbon yield” dataset can be used to analyse spatially distributed net loss and net gain of riverine organic carbon over the conterminous United States. For example, users can analyse what are the major factors determining reach-scale carbon net loss or net gain.
4. Despite recent emphasis on the importance of riverine carbon budgets over the CONUS, there is still a lack of spatial information regarding net gain/loss of riverine carbon. Therefore, the “watershed carbon yield” dataset could be used as a component of future synthesis efforts that are aimed at accounting for the riverine carbon budgets over the CONUS. For example, we combine the “watershed carbon yield” dataset with other riverine carbon cycling datasets^31^ to improve riverine carbon budgeting.
5. Both the “POC/DOC load at hydrologic stations” and “watershed carbon yield” can be further combined with global scale riverine carbon dataset (such as the riverine carbon load compiled by Wohl *et al*.^22^ and GEMS/WATER Global Register of River Inputs (GEMS-GLORI) database^7^) to support global scale riverine carbon analysis. It is worth noting that both “POC/DOC load at hydrologic stations” and “watershed carbon yield” represent estimates of historical carbon load and yield. The algorithms used here do not provide predictive capability to estimate carbon load at ungauged locations. The “watershed carbon yield” was estimated dividing differences in carbon load between upstream and downstream hydrological stations by the area controlled by those hydrological stations, thereby not considering the underlying complex terrestrial and aquatic processes regulating carbon yield in different watersheds. Future studies could explore using machine learning and other techniques and datasets^32^ to further leverage the datasets to predict carbon load and yield in ungauged basins.

## Data Availability

All the codes for processing the NHDplus data and generating the watershed carbon yield maps were developed using MATLAB version 2020b and archived at Github: https://github.com/qhgogogo/Spatially-distributed-riverine-organic-carbon.
